# Trace Elements and Temperature Combined to Regulate Zooplankton Community Structures in Mountain Streams

**DOI:** 10.3390/biology14020183

**Published:** 2025-02-11

**Authors:** Li Ji, Huayong Zhang, Zhongyu Wang, Yonglan Tian, Wang Tian, Zhao Liu

**Affiliations:** 1 Research Center for Engineering Ecology and Nonlinear Science, North China Electric Power University, Beijing 102206, China; geelyz@163.com (L.J.); zhy_wang@ncepu.edu.cn (Z.W.); yonglantian@ncepu.edu.cn (Y.T.); tianwang@ncepu.edu.cn (W.T.); 2 Theoretical Ecology and Engineering Ecology Research Group, School of Life Sciences, Shandong University, Qingdao 250100, China; liuzhao9555@sdu.edu.cn

**Keywords:** mountain stream, zooplankton community, temperature, trace element, community structure, synergistic effect

## Abstract

Zooplankton, as an important component of mountain river ecosystems, play a crucial role in the cycling of matter and the flow of energy. However, the depth and systematic research on the formation mechanisms and influencing factors of zooplankton communities in mountain streams are poorly understood. Here, we conducted field sampling and investigated the spatiotemporal distribution of the zooplankton community structure and the major environmental factors in mountain streams to explore the principles underlying their effects on the zooplankton community. The results showed that zooplankton community structure exhibited significant seasonal variations. Rotifera and Cladocera were the dominant groups, with Rotifera dominant in warm weather and Cladocera dominant in February. The analysis revealed that temperature and trace elements are the main factors affecting zooplankton diversity. Our model explained 46.50% of the variation in zooplankton through temperature, water properties, nutrients, and trace elements. The results highlighted that temperature acted directly on the zooplankton community and also exhibited indirect and negative effects on zooplankton diversities through altering trace elements. Trace element variables had a significant impact on zooplankton community distribution. Our study systematically quantified these relationships, providing insights into the ecological processes of mountain streams and offering a scientific basis for the ecological protection of mountain streams.

## Simple Summary

Zooplankton play a crucial role in the cycling of matter and the flow of energy. However, the depth and systematic research on the formation mechanisms and influencing factors of zooplankton communities in mountain streams are poorly understood. Here, we conducted field sampling and investigated the spatiotemporal distribution of the zooplankton community structure and the major environmental factors in mountain streams to explore the principles underlying their effects on the zooplankton community. The results showed that zooplankton community structure exhibited significant seasonal variations. Rotifera and Cladocera were the dominant groups, with Rotifera dominant in warm weather and Cladocera dominant in February. The analysis revealed that temperature and trace elements are the main factors affecting zooplankton diversity. Our model explained 46.50% of the variation in zooplankton through temperature, water properties, nutrients, and trace elements. The results highlighted that temperature acted directly on the zooplankton community and also exhibited indirect and negative effects on zooplankton diversities through altering trace elements. Trace element variables had a significant impact on zooplankton community distribution. Our study systematically quantified these relationships, providing insights into the ecological processes of mountain streams and offering a scientific basis for the ecological protection of mountain streams.

## 1. Introduction

Mountain rivers, as an important part of freshwater ecosystems, although they cover a relatively limited area on the Earth’s surface, play an indispensable role in regional and even global ecological balance due to their unique geographical environment and ecological characteristics [1]. Mountain rivers usually have characteristics such as rapid water flow, large drops, and significant changes in water temperature with altitude, which have nurtured rich and unique biodiversity [2,3]. Zooplankton, as an important component of mountain river ecosystems, play a crucial role in the cycling of matter and the flow of energy [4]. They are the main consumers of phytoplankton, effectively controlling the quantity and distribution of phytoplankton. At the same time, they were also an important food source for many higher trophic level organisms such as fish and amphibian larvae. The changes in their community structure and biomass were directly related to the stability of the entire food chain and the function of the ecosystem [5,6].

With global climate change, mountain river ecosystems are facing unprecedented challenges. Global warming has led to a significant upward trend in the water temperature of mountain rivers, and this change has had multiple impacts on the physiological and ecological processes of zooplankton [7]. Previous studies have shown that rising temperatures can accelerate the metabolic rate of zooplankton, thereby affecting their growth, development, and reproductive cycles [8]. Within the appropriate temperature range, an increase in temperature may promote the reproduction of zooplankton and boost their population numbers. However, it may cause physiological disorders in zooplankton and even lead to death when the temperature exceeds a certain threshold. At the same time, the interference of human activities in mountain river ecosystems was also increasing [9,10]. Activities such as the development of industrialization and agriculturalization around mountainous areas have significantly changed the content and composition of trace elements in mountain rivers. Trace elements such as iron, zinc, copper, and manganese, although their contents in water bodies were relatively low, were crucial for the life activities of zooplankton [11]. Appropriate amounts of trace elements were important components of many enzymes and proteins in zooplankton, participating in physiological processes such as metabolism, growth and development, and immune regulation [12].

Early studies about zooplankton mostly focused on the impact of single factors such as temperature or trace elements on them. With the deepening of research, more and more scholars have realized that in the natural environment, temperature and trace elements do not act in isolation, but there are complex synergistic effects [13,14]. Zheng et al. (2025) found that within a certain range of temperature and trace element concentrations, the synergistic effect of the two can promote the growth and reproduction of zooplankton, increasing the biomass and diversity of the community. However, when the temperature is too high or the trace element concentration exceeds the appropriate range, this synergistic effect will have an inhibitory effect on the zooplankton community, resulting in the simplification of the community structure and the decline of biomass [15]. Studies have shown that the interaction between temperature and trace elements can significantly affect the enzyme activity in zooplankton through feeding experiments on specific zooplankton species and then affect their feeding, metabolism, and energy conversion efficiency [16]. There are also studies that use a combination of field investigations and laboratory experiments to explore the key role of trace element bioavailability in the impact of temperature changes on the zooplankton community [17]. Zhang et al. (2022) pointed out that temperature changes can affect the existing forms and bioavailability of trace elements in water bodies, and the uptake and utilization of trace elements by zooplankton depend to a large extent on their bioavailability [17]. Although certain progress has been made in the research on the synergistic effects of temperature and trace elements on the zooplankton community in freshwater ecosystems, there are still many deficiencies in the research on the zooplankton community in mountain rivers. Currently, the comprehensive action mechanisms between temperature, trace elements, and these special environmental factors are still unclear, and there are large differences in the weight distribution of multiple factors in different studies, which brings great difficulties in accurately predicting and assessing the impact of environmental changes on the zooplankton community in mountain rivers. In addition, existing studies have certain limitations in the degree of fit between the experimental environment and the natural environment of mountain rivers and the comprehensiveness of the research scope.

This study aims to investigate the synergistic effects of temperature and trace element fluctuations on the zooplankton community in mountain rivers. By using field investigations and advanced analytical techniques, we will analyze their synergistic effects in more depth. Specifically, we conducted and collected field investigations in the Taizicheng River ecosystem to analyze the zooplankton community, environmental factors, and trace element contents. We explored the complex relationships between temperature, trace elements, other environmental factors, and the zooplankton community to shed light on their comprehensive impacts on the zooplankton community. It is expected to offer new ideas and methods for addressing the challenges posed by global climate change and human activities to mountain river ecosystems.

## 2. Materials and Methods

### 2.1. Study Area

**Figure 1:**
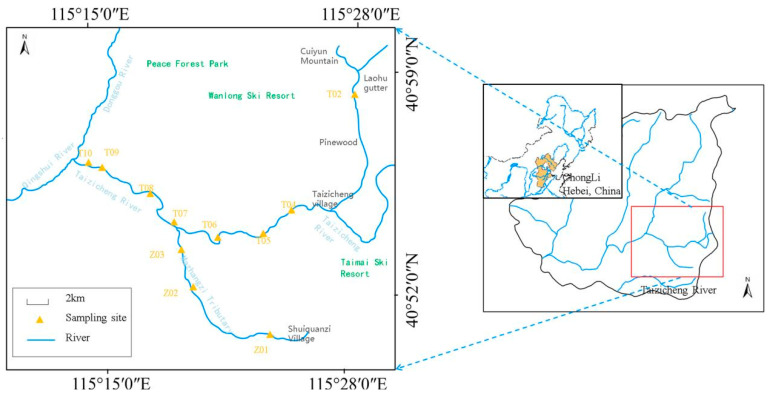
Location of the Taizicheng River and sampling sites.

Taizicheng River, located in the heart of the Chongli District in Zhangjiakou, China, is a mountain river of significant importance, particularly as a crucial part of the 2022 Winter Olympics’ core zone (Figure 1). The river originates from densely forested mountains, with its basin surrounded by mountains with a vegetation coverage exceeding 65%. The dominant forest types in the region are *Betula platyphylla Suk* and *Larix thibetica*. In the upper reaches of the river, the dense vegetation near the riverbanks provides shade, lowering the water temperature in the adjacent areas. The roots of the riverside plants offer attachment points for zooplankton. The river spans a length of 30.50 km, with an average width of approximately 2.00 m. The general depth is less than 1.00 m, and river transparency ranges from 0.01 to 0.70 m (Table S1). Such a river channel and water depth contribute to the formation of turbulent flow, enhancing the mixing of water, nutrients, and oxygen. The river, with an altitude from 1180 m to 1910 m and rapid flow, lies in a region of typical continental monsoon climate: cool, humid summers and long, cold winters. The average temperature in summer is around 19 °C and averages −12 °C in winter. The river’s flow velocity ranges from 0.06 to 1.96 m/s. The seasonal changes in precipitation patterns directly affect the stream’s discharge. During the rainy season, the stream has a larger water volume and faster flow. In winter, when the area experiences freezing and snowfall, as precipitation turns into snow, the mountain runoff decreases significantly, causing the river’s water volume to decline. The cold temperature slows down the water flow, thus affecting the distribution and movement of zooplankton. The freezing and snowfall period in the region lasts from November to March. The area receives approximately 500 mm of rainfall annually. Mountain runoff following precipitation is the primary water source for the river. In the upper reaches of the Taizicheng River, the water flow is rapid, and the substrate mainly consists of gravel and organic debris sediments. In the middle and lower reaches, the flow rate is relatively slow, and the substrate is mainly sand or silt sediments. The river is abundant in nutrients such as nitrogen and phosphorus, and the concentrations of these nutrients display substantial seasonal fluctuations.

### 2.2. Sampling and Environmental Variables

A total of 11 sampling sites were established along the river (Figure 1). At the first tributary (Mazhangzi), Sites Z01–Z03 were set. At the second tributary, Sites T02–T06 were designated. Along the river’s mainstream, T07–T10 were set up. All investigations and measurements were conducted quarterly from May 2020 to February 2021 (May 2020, August 2020, November 2020, and February 2021). Water temperature, pH, and total dissolved solids (TDS) were measured in situ using a multiparameter sonde (YSI Professional Plus, YSI Group, Ohio, USA). Water depth and transparency were measured using a hand-waving tape measure with a Secchi disk. Vertically and slowly lower the Secchi disk into the water. Read the scale of the measuring rope at the water surface when the Secchi disk just disappears from sight. Conduct three measurements at the same point and take the average value as the water transparency at that point. Data showed that transparency values fluctuated within a certain range. Water flow velocity was measured at the river’s surface using a SonTek FlowTracker2 (Summers Ridge Rd, San Diego, CA, USA). At each site, two 1 L surface water samples were collected in previously cleaned polyethylene bottles: one for element analyses and the other for physicochemical analyses. The water samples were sent to the laboratory within 24 h to determine physicochemical factors and elements. For zooplankton samples, we used a No. 25 plankton net (64 μm, for Protozoa Goldfuss, 1818, and Rotifers) and a No. 13 plankton net (112 μm, for Crustaceans) for horizontal drag sampling to avoid omissions of different taxa due to size differences. Sampling was repeated three times at each sampling point. Since the stream is shallow, we did not set multiple vertical sampling points. To ensure consistent sampling effort, the towing time was kept largely the same each time. Samples were immediately preserved with 5% acidic Lugol’s solution, settled for 24 h at room temperature, and siphon-concentrated into 50 mL amber bottles. For the main zooplankton groups (Rotifera Cuvier, 1798; Cladocera Latreille, 1829; and Copepoda Milne-Edwards, 1840), classification and counting were carried out using an inverted microscope (OPTIKA IM-3, M.A.D. Company, Ponteranica, Italy), and morphological identification was referred to the Atlas of Freshwater Plankton in China. Protozoa Goldfuss, 1818 were pre-fixed with 3% glutaraldehyde to maintain the integrity of the ciliary structure, and quantitative analysis was carried out using the Utermöhl sedimentation chamber method. All samples were indeed documented. We used a digital data entry system to record the abundance of zooplankton in each sample.

The chemical oxygen demand (COD), ammonia nitrogen (NH_3_-N), total nitrogen (TN), total phosphorus (TP), nitrogen (NO_3_-N), and phosphoric acid (PO_4_) were analyzed in the laboratory based on the National Standard of China (GB3838-2002) (https://www.codeofchina.com/standard/GB3838-2002.html) [18]. The copper (Cu), calcium (Ca), magnesium (Mg), iron (Fe), manganese (Mn), zinc (Zn), chromium (Cr), kalium (K), silicon (Si), arsenic (As), nickel (Ni), molybdenum (Mo), boron (B), and cobalt (Co) were determined by inductively coupled plasma mass spectrometer (ICP-MS) (Agilent 7800, Heng Sheng Mass Spectrometer Company, Shanghai, China). The C and S were determined by an element analyzer (Vario EL cube, Elementary Corporation, Langenselbold, Germany).

### 2.3. Statistical Analyses

We separately analyzed the total sample datasets to assess variations in zooplankton communities and environmental factors in four months. Box plots with statistically significant differences based on Kruskal–Wallis H-tests were performed to illustrate the four moths’ variations in environmental factors by using the vegan package in R version 4.2.2. Diversity and community composition analyses of the zooplankton community were performed using the phyloseq package [19]. To visualize the relative abundance of phytoplankton groups and the most abundant species in the community, stacked bar plots were created at the phylum and species levels using the “transform_sample_counts” function. Circos plots were used to show the seasonal changes in zooplankton at the phylum level (https://www.bioincloud.tech, accessed on 20 December 2024). Non-metric multidimensional scaling (NMDS) was conducted based on the Bray–Curtis distance matrices to visualize the composition dissimilarities of zooplankton. Alpha diversity was measured using the “estimate_richness” function in phyloseq. Three biodiversity indices, namely species richness, Shannon-Wiener diversity, and Pielou’s evenness, were investigated. An upset plot was used to visualize the size and distribution of zooplankton species intersections between groups by using the UpSetR package (1.6.0).

Environmental factors with a variance inflation factor (VIF) of less than 10 were selected for Canonical Correlation Analysis (CCA) to explain the effects of multiple factors on variations in zooplankton species by using Canoco 5.0. To assess the independent contribution of each environmental variable to the total variability in zooplankton, we performed a Hierarchical Partitioning (HP) analysis using the rdacca.hp package in R version 4.2.2 [20]. Significant testing for independent contributions was not conducted in HP analysis, as predictor importance was generally regarded as an exploratory framework for interpreting regression, rather than an inferential tool. Variation partitioning analysis (VPA) was further employed to examine the relative importance of trace elements, physical and chemical properties, and nutrients in shaping the zooplankton by using the varpart function in the “vegan”.

Additionally, a Mantel test based on Spearman’s rank correlation was used to determine the relationships between zooplankton biomass or diversity and environmental variables. A heatmap was created to visualize the relationships among environmental variables, and the analyses were conducted using the vegan, corrplot, and dplyr packages in R version 4.2.2 [21]. Finally, we used partial least squares path modeling (PLS-PM) to show the relationships between zooplankton community structure, nutrients, trace elements, and temperature. Non-significant paths were removed from the model. The goodness-of-fits (GoF), which measured the overall predictive power of the model, was calculated using partial least squares path modeling in the plspm package in R version 4.2.2 [22,23].

All statistical analyses were conducted using R 4.2.2 and visualized with the “ggplot2” package in R 4.2.2 [24].

## 3. Results

### 3.1. Variations in Water Physicochemical Parameters

**Figure 2:**
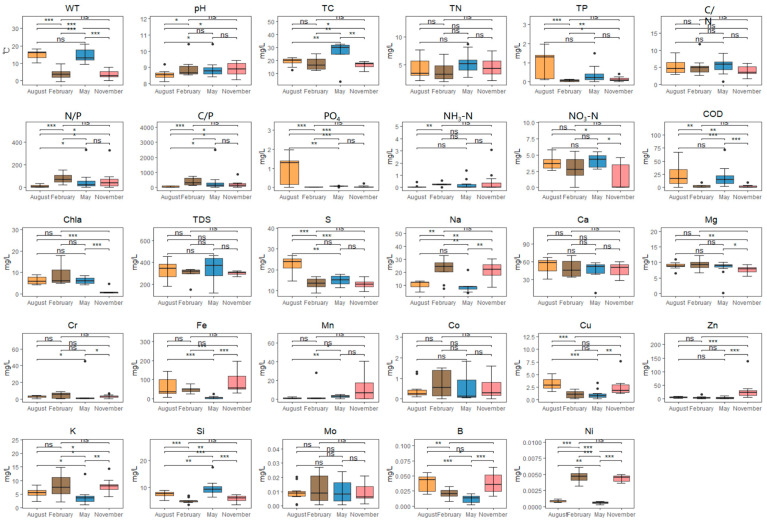
The temporal variation in water quality indicators. A significant correlation was confirmed if the *p*-value was < 0.05 (*), 0.01 (**), 0.001 (***) and insignificance (ns).

The water quality indicators of Taizicheng River in four months were summarized (Figure 2). Overall, the annual water temperature variation range of the Taizicheng River was −0.60~21.10 °C (*p* < 0.001), the water pH in the Taizaicheng River was alkaline, there was no significant difference in TDS and pH among the months, while the concentration of Chla and COD showed significant month differences, with the concentration being lower in February (*p* < 0.001) (Figure 2). TP and PO_4_ concentrations were similar and varied between 0 and 1.97 mg/L. The TP, PO_4_, and S concentrations in August were significantly higher than those of other seasons (*p* < 0.001) (Figure 2). Significant differences were observed among the trace elements Na, Fe, Cu, Zn, Si, B, and Ni. The Na, K, and Ni concentrations in February were significantly higher *(p* < 0.001), while Fe, Cu, and B concentrations in August were significantly higher (*p* < 0.001).

### 3.2. Community Composition and Diversity Characteristics of Zooplankton

**Figure 3:**
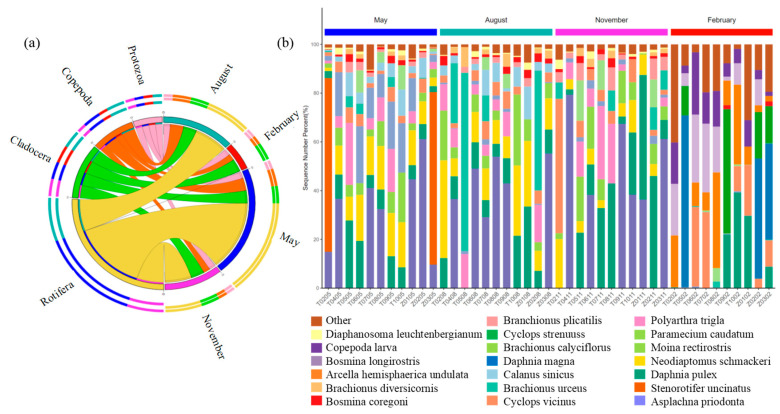
(**a**) The string diagram shows the distributions of zooplankton compositions at the phylum level in four months. Rotifera Cuvier, 1798, is shown in yellow; Cladocera Latreille, 1829, is shown in blue; Copepoda Milne-Edwards, 1840, is shown in orange; and Protozoa Goldfuss, 1818, is shown in pink. (**b**) Histograms showing the zooplankton community compositions on the genus level at each site.

A total of 52 zooplankton species were identified and assigned into four phyla, namely Rotifera Cuvier, 1798, Cladocera Latreille, 1829, Copepoda Milne-Edwards, 1840, and Protozoa Goldfuss, 1818 (Figure 3). In general, Rotifera Cuvier, 1798, and Cladocera Latreille, 1829, comprised the majority of the zooplankton phyla. From May to February, the relative abundance of Rotifera Cuvier, 1798, was 68.57%, 49.10%, 52.72%, and 1.08%, respectively, while the relative abundance of Cladocera Latreille, 1829, was 12.78%, 20.68%, 30.28%, and 46.15%, respectively. Rotifera Cuvier, 1798, dominated in May followed by November and August, but it barely appeared in February, suggesting that it may not have been adapted to survive in very cold environments like in February. Cladocera Latreille, 1829, was most abundant in February, suggesting that it may play an important metabolic role in this environment. Copepoda Milne-Edwards, 1840, and Protozoa Goldfuss, 1818, were relatively low in abundance in all four environments, where they were not a major community member and may play a secondary role (Figure 3a). Figure 3b exhibited the variations in zooplankton compositions at the genus level in four months at each site. Overall, *Asplachna priodonta Gosse, 1850,* was the predominant genus throughout the year, with its relative abundance of 19.79% in May, 32.30% in August, 37.09% in November, and 0% in February. *Stenorotifer uncinatus Milne, 1886*, was the second dominant genus, which only existed in May and had an abundance of 37.96%. The distribution of species at each site was very similar in May, August, and November. However, the distribution of species changed dramatically in February. *Asplachna priodonta Gosse, 1850*, *Stenorotifer uncinatus Milne, 1886*, *Neodiaptomus schmackeri (Poppe & Richard, 1892)*, *Moina rectirostris (Leydig, 1860)*, and *Paramecium caudatum Ehrenberg, 1838,* disappeared, and *Daphnia magna Straus, 1820*, *Cyclops strenuus Fischer, 1851*, and *Arcella hemisphaerica undulata Deflandre, 1928*, appeared at the same time in February.

**Figure 4:**
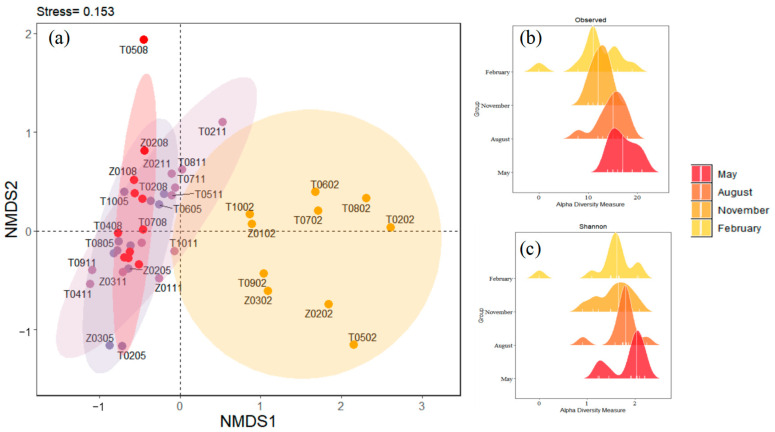
(**a**) The non-metric multidimensional scaling (NMDS) shows the compositions of zooplankton taxa in four months. The solid dot indicates zooplankton communities at each sampling site in each month. Different colors indicate different months: modena for May, red for August, lilac for November, and yellow for February. (**b**) Observed index in Taizicheng River over four months. (**c**) Shannon index in Taizicheng River over four months.

Non-metric multidimensional scaling (NMDS) was performed to explore the composition of zooplankton taxa across the four months. Significant segregations between February and the other three months’ sampling groups were found for zooplankton (Figure 4a). This indicated that the zooplankton community structure in February was significantly different from those in May, August, and November. The distribution range of samples in the February group was large, indicating that the community structure of samples in the February group was very different. The samples of the May group, August group, and November group overlapped to some extent, indicating that the three groups had some similarities in community composition. Regarding the alpha diversity of zooplankton taxa (Figure 4b,c and Figure S1), the observed richness (17.09 ± 2.59) and Shannon diversity (1.86 ± 0.37) were significantly higher in May than in the other three months, and the observed was the lowest in February (12.08 ± 3.29), whereas evenness was not significantly different in the four months (*t*-test, *p* < 0.001).

**Figure 5:**
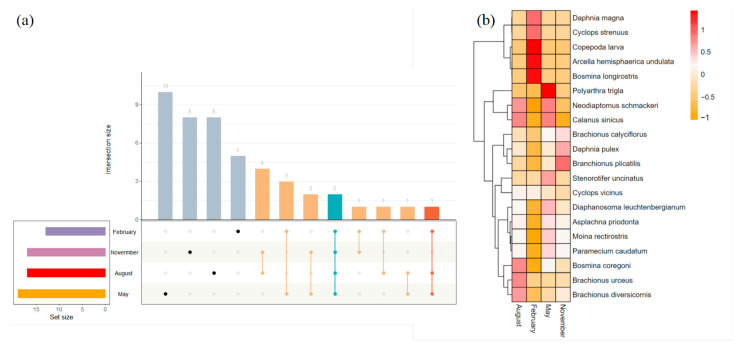
(**a**) Upset diagram depicting the number of shared and unique species in the stream during the four months and (**b**) distribution of the zooplankton species among the four months.

Among all zooplankton species sampled, two species were present in all four months (Figure 5a). Additionally, there were 10 specifics to the May samples, 8 specifics to the August samples, 8 specifics to the November samples, and 5 specifics to the February samples. *Polyarthra trigla Ehrenberg, 1834*, dominated the zooplankton community in May. In November, the proportion of *Brachionus plicatilis Müller, 1786*, increased, and Copepoda larvae dominated the zooplankton community in February (Figure 5b). This showed that the community of species in February was significantly changed compared to the other three months.

### 3.3. Driving Factors for Zooplankton Communities

**Figure 6:**
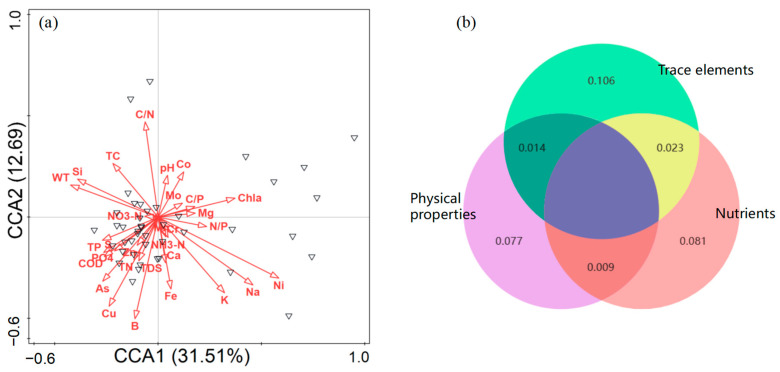
(**a**) Redundancy analysis showing the effects of multiple factors on zooplankton taxa, black triangle represents each sample and (**b**) variation partitioning analysis displaying the explanation percentages for zooplankton at the species level. Physical properties (purple): WT, pH, COD, Chla, and TDS. Nutrients (pink): TC, TN, TP, C/N, C/P, N/P, S, NH_3_-N, and NO_3_-N. Trace elements (green): Na, Ca, Mg, Cr, Fe, Mn, Co, Cu, Zn, K, Si, Mo, B, Ni, and As.

We revealed the contributions of influencing factors in affecting zooplankton by using the RDA analysis (Figure 6a). The first two constraint axes were also meaningful at *p* < 0.01 and proved using Monte Carlo permutation tests (999 permutations) with Bonferroni adjustment. They explained 44.20% of the total variance in the zooplankton abundance; axis one explained 31.51% and axis two explained 12.69%. Among the multiple influencing variables, Chla (10.80%), Ni (10.00%), B (3.80%), WT (2.70%), K (2.60%), and N/P (2.50%) were the main determinants of zooplankton (Figure S2). In addition, variation partitioning results suggested that a greater percentage of zooplankton variations could be explained by purely trace element variables (10.60%) rather than purely physical properties (7.70%) or nutrients (8.10%) (Figure 6b). These results indicated that trace element variables had a higher influence on community distribution.

**Figure 7:**
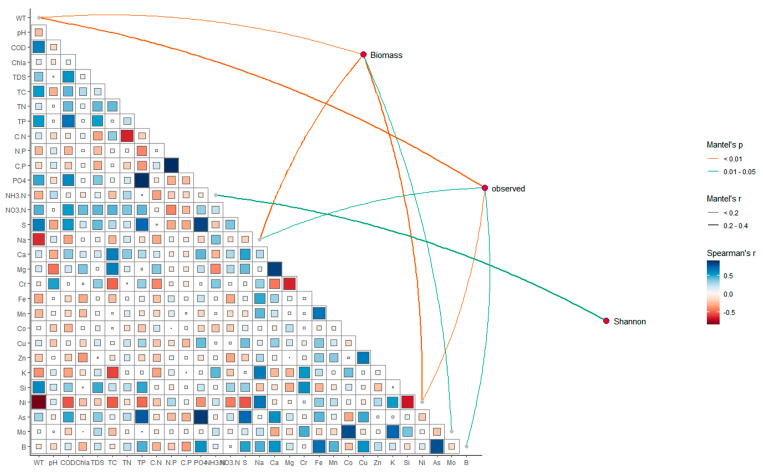
Correlations between zooplankton and environmental variables by Mantel tests. Edge width represents the R-value, orange lines represent significance *p*-value < 0.01, and green lines represent significance 0.01 < *p*-value < 0.05, and edge color indicates a statistically significant color gradient that corresponds to Spearman correlation coefficients between environmental indicators.

Through the Mantel tests (Figure 7), the connections between zooplankton communities and environmental factors were displayed, and the specific information was listed in Table S2. A significant correlation was observed between the contents of Na (*r*^2^ = 0.22, *p* < 0.05), Ni (*r*^2^ = 0.30, *p* < 0.01), and WT (*r*^2^ = 0.15, *p* < 0.01) with the zooplankton biomass. The observed zooplankton communities showed a significant correlation with concentrations of Ni (*r*^2^ = 0.19, *p* < 0.01), Na (*r*^2^ = 0.12, *p* < 0.05), B (*r*^2^ = 0.15, *p* < 0.05), and WT (*r*^2^ = 0.23, *p* < 0.01), while the Shannon of zooplankton communities showed a significant correlation with NH_3_-N (*r*^2^ = 0.34, *p* < 0.01). Figure 6 also exhibited the correlations among different water quality parameters. Specifically, COD, Na, and Ni were significantly correlated with WT. The S and As were strongly correlated with PO_4_. These results suggested there were various correlations among different water quality parameters, which may influence the zooplankton through synergistic or direct ways.

**Figure 8:**
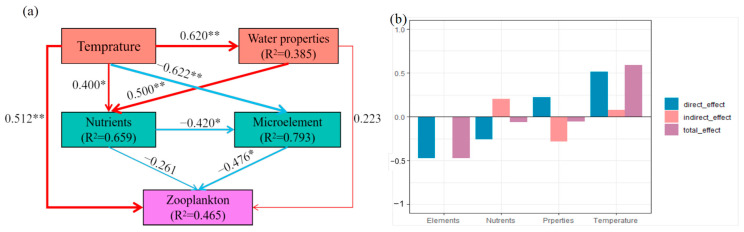
(**a**) Partial least squares path modeling (PLS-PM) revealed the direct effects of environmental variables on zooplankton based on temperature. All environmental data were log (x + 1) transformed. Water properties: pH, COD, and TDS; nutrients: TC, TP, NO_3_-N, PO_4_, and S; trace elements: Na, Mg, Ca, Fe, Zn, K, Si, Ni, and As; zooplankton: observed, Shannon, and chao1. The widths of arrows and lines represent the magnitude of path coefficients, and the blue lines and red lines indicate the positive and negative effects, respectively (significance: * *p* < 0.05, ** *p* < 0.01). The goodness-of-fit (GoF) was 0.51. (**b**) Standardized effects on zooplankton from PLS-PM based on temperature in Taizicheng River.

The separate models based on temperature (Figure 8a) were built to explore the influences of environmental variables on the zooplankton diversities, and the specific effects were exhibited in Figure 8b. Our partial least squares-path model explained 46.50% of the variation in zooplankton through temperature, water properties, nutrients, and trace elements (Figure 8). Temperature was the most significant factor affecting the zooplankton diversity, and the direct effect was 0.51 (*p* < 0.01). Temperature significantly affected water properties, trace elements, and nutrients, with a direct effect of 0.62 (*p* < 0.01), −0.62 (*p* < 0.01), and 0.40 (*p* < 0.01), respectively. Furthermore, both trace elements and nutrients had a direct negative effect on zooplankton diversities, with −0.48 (*p* < 0.01) and −0.26 (*p* < 0.01), respectively. More importantly, temperature also exhibited indirect and negative effects on zooplankton diversities through altering trace elements.

## 4. Discussion

Temperature changes can alter the physical structure of habitats and hydrological properties in aquatic ecosystems [25]. The effects of trace elements and nutrients on zooplankton diversities can adjust the delicate balance required for a healthy zooplankton community [26]. Our present study investigated the interaction between the environment and zooplankton and uncovered several important aspects regarding the factors influencing zooplankton communities in the Taizicheng River of the northern mountainous area in China. This comprehensive understanding of these relationships contributes to the fundamental knowledge of how mountain stream ecosystems function, especially in terms of the factors governing the diversity of key components like zooplankton.

### 4.1. Variations in Zooplankton Community

A total of 52 zooplankton species were identified and classified into four phyla in this study. Rotifera Cuvier, 1798, and Cladocera Latreille, 1829, accounted for the majority, which is consistent with the research results of other freshwater ecosystems [27]. These two types of zooplankton usually have strong reproductive capabilities and environmental adaptability. They can quickly respond to environmental changes and utilize abundant resources for population growth. Rotifera Cuvier, 1798, are small in size and have a fast reproduction rate, enabling them to rapidly expand their population in suitable environments. On the other hand, Cladocera Latreille, 1829, has an efficient filtering ability, which allows them to better compete for food resources [27,28]. Brackish-water species such as Brachionus plicatilis and Diaphanosoma leuchtenbergianum were found in our study area, likely due to multiple factors. These euryhaline species can tolerate the seasonal variations in the Taizicheng River, a nitrogen and phosphorus-rich freshwater environment shaped by its complex ecosystem and typical continental monsoon climate, thanks to their physiological adaptability. Situated in the core area of the 2022 Winter Olympics, the river may experience salt release and changes in water chemistry from related construction, operation, and local human activities, creating microhabitats favorable for these species. Moreover, the river may be connected to other water systems, allowing brackish-water species to enter via underground currents or runoff. Migratory activities of birds and other aquatic animals may also transport their eggs or larvae. From May to February, the relative abundances of Rotifera Cuvier, 1798, and Cladocera Latreille, 1829, showed obvious seasonal variations. Rotifera Cuvier, 1798, dominated in warm months but almost disappeared in February. Some studies have pointed out that the growth and reproduction of Rotifera Cuvier, 1798, are extremely sensitive to temperature, and the suitable temperature range is usually between 15 and 25 °C [29]. In warm seasons, the water temperature is suitable, and phytoplankton grow vigorously, providing an abundant food source for Rotifera Cuvier, 1798, and promoting their rapid reproduction. However, the low temperature in mountain rivers may inhibit the metabolism and reproductive ability of Rotifera Cuvier, 1798, in February and even cause the death of some individuals, leading to a sharp decline in their population. Some Cladocera Latreille, 1829, species have special low-temperature adaptation mechanisms and can adjust their metabolic pathways to maintain life activities in low-temperature environments. In addition, the low-temperature environment may reduce the activities of Cladocera Latreille, 1829, natural enemies, reducing the predation pressure and facilitating the growth of their population [30]. Copepoda Milne-Edwards, 1840, and Protozoa Goldfuss, 1818, had relatively low relative abundances in four months. This may be because their ecological niches in the mountain river ecosystem are relatively narrow, and their adaptability to environmental changes is weak. Yi et al. (2024) mentioned that Copepoda Milne-Edwards, 1840, and Protozoa Goldfuss, 1818, are at a disadvantage in resource competition with Rotifera Cuvier, 1798, and Cladocera Latreille, 1829, resulting in difficulty in a significant increase in their population size [31]. *Asplachna priodonta Gosse, 1850*, was dominant for most of the year but disappeared in February. *Stenorotifer uncinatus Milne, 1886*, only existed in May and had a high abundance. This may be related to the special requirements of these species for temperature and food resources. The research has found that *Asplachna priodonta Gosse, 1850*, prefers higher temperatures and specific types of phytoplankton as food [32]. In February, the low temperature may have changed the types and quantities of phytoplankton in the water body, making it unable to meet the survival needs of *Asplachna priodonta Gosse, 1850*, resulting in its disappearance.

The NMDS showed that the zooplankton community structure in February was significantly different from that in the other three months, and the distribution range of samples in February was large. In February, there were significant changes that occurred in the species distribution. This indicates that the environmental factors in February, such as temperature, trace elements, and food resources, were different from those in other months, resulting in a unique and more dispersed zooplankton community structure. The low temperature might have altered the chemical properties of the water body, affected the solubility and bioavailability of trace elements, and thus influenced the living environment of zooplankton. At the same time, the changes in food resources also prompted adjustments in the zooplankton community structure [14,33]. Temperature and food resources are important factors influencing zooplankton diversity [34,35]. In May, it was conducive to the survival and reproduction of different types of zooplankton with suitable temperatures and abundant food resources, thereby increasing species richness and diversity. However, the low-temperature environment in February may have restricted the survival of many zooplankton, leading to a decrease in diversity. There was no significant difference in the evenness of the zooplankton community over the four months, indicating that the relative abundance distribution of zooplankton was relatively uniform each month.

### 4.2. Temperature and Trace Elements as the Primary Drivers of Variations in Zooplankton Community

In our study, the results of redundancy analysis and variation partitioning indicated that among multiple influencing variables, chlorophyll a (Chla), nickel (Ni), boron (B), potassium (K), and water temperature (WT) were the main determinants of zooplankton, with trace element variables having a greater impact on community distribution. Many studies have confirmed the significant influence of environmental factors on zooplankton communities [36]. Water temperature (WT), as an important environmental factor, not only directly affects the physiological activities of zooplankton but also indirectly impacts the zooplankton community by influencing other environmental variables. As discussed previously, the changes in water temperature can affect the metabolic rate, reproductive capacity, and food intake efficiency of zooplankton [37]. Chlorophyll a, as an indicator of phytoplankton biomass, is directly related to the food supply of zooplankton. A higher content of chlorophyll usually implies more abundant food resources, which is beneficial for the growth and reproduction of zooplankton [38]. The effects of trace elements such as Ni and B on zooplankton are relatively complex. On the one hand, appropriate amounts of trace elements are important components of many enzymes and proteins in zooplankton, participating in physiological processes such as metabolism, growth and development, and immune regulation [11]. On the other hand, when the concentration of trace elements in rivers is too high or too low, it may have a negative impact on zooplankton. In this study, the significant impact of trace element variables on the distribution of the zooplankton community may be due to the unique geological and hydrological conditions of the Taizicheng River, such as fast flowing, large temperature differences, and large gradients, which lead to changes in the content and form of trace elements, thus affecting the survival and distribution of zooplankton [39]. K and N/P also have an important impact on the zooplankton community. The concentration and proportion of nutrients can affect the growth and species composition of phytoplankton and thus influence the food quality and quantity of zooplankton [40]. In this study, although the proportion of variation in zooplankton explained by nutrients was relatively low, they still remain one of the factors affecting the zooplankton community.

Our results showed that Na, Ni, and WT were significantly correlated with the zooplankton biomass, and there were various correlations among different water quality parameters. Some studies analyzed the impacts of the synergistic effects among environmental factors on the zooplankton community through the Mantel test, indicating that environmental factors (including trace elements, water temperature, and nutrients) affect the zooplankton community either directly or synergistically, which is consistent with the results of this study [41]. Our path model found that temperature is the most important factor directly affecting zooplankton diversity, which is in line with this general understanding. Both trace elements and nutrients have a direct negative impact on zooplankton diversity. More importantly, temperature also has an indirect negative impact on zooplankton diversity by altering trace elements, depicting a complex interaction network. The study has shown that temperature plays a central role in aquatic ecosystems [42]. The changes in temperature can affect the physicochemical properties of water bodies, such as dissolved oxygen content, pH value, and so on, thus influencing the living environment of zooplankton [43]. In this study, temperature has a significant direct impact on water quality, which may be because temperature changes can affect the gas solubility and chemical reaction rates in water, thereby changing water quality parameters. The negative impact of temperature on trace elements may be due to the fact that an increase in temperature causes changes in the chemical forms of certain trace elements, reducing their bioavailability or accelerating the precipitation and loss of trace elements [44]. The direct negative impact of trace elements on zooplankton diversity may be because either too high or too low concentrations of trace elements can interfere with the physiological processes of zooplankton, such as affecting their enzyme activity, energy metabolism, and reproductive functions [45,46,47].

Our study can advance the understanding of the ecological processes of zooplankton communities in mountain streams, providing deeper insights into the relative importance of different environmental factors in shaping these communities. Understanding how changes in temperature and trace elements affect zooplankton is conducive to formulating targeted conservation strategies. It not only provides a theoretical basis for the protection of mountain river ecosystems but also contributes to water resource management and water quality monitoring, offering a reference for addressing the impacts of climate change on mountain rivers. However, our study has some limitations, such as a short sampling period and the failure to consider other influencing factors. In future research, long-term monitoring programs should be carried out, and additional variables (such as predator abundance and quantity and quality of organic matter) should be measured. The research focus should be placed on understanding the interactive effects of temperature, trace elements, and other stressors (such as chemical pollutants and habitat fragmentation) on zooplankton communities in mountain streams. To advance the understanding of zooplankton navigation mechanisms in such ecosystems, we consider future studies exploring the specific signals and mechanisms used by zooplankton for efficient navigation by using advanced imaging techniques. This will enable us to comprehensively understand the behavior of zooplankton in real time and how these organisms respond to multiple environmental changes.

## 5. Conclusions

By delving into the relationships between zooplankton communities, temperature, and trace elements in mountain rivers, we found that there were a total of 52 zooplankton species belonging to four phyla, among which Rotifera Cuvier, 1798, and Cladocera Latreille, 1829, were in the majority. Zooplankton community structure exhibits a significant seasonal variation. Rotifera Cuvier, 1798, dominated in warm months, while Cladocera Latreille, 1829, was most abundant in February. Copepoda Milne-Edwards, 1840, and Protozoa Goldfuss, 1818, had relatively low abundances throughout the year. Through methods such as NMDS, RDA, variation partitioning, Mantel test, and PLS-PM, the impacts of multiple environmental factors on zooplankton have been revealed. Our results indicated that temperature was the most significant factor affecting zooplankton diversity. It not only acted directly on zooplankton but also exerted indirect effects by influencing water quality, trace elements, and nutrients. Both trace elements and nutrients directly negatively impacted zooplankton diversity, and trace element variables had a relatively large impact on the distribution of the zooplankton community. Our study systematically quantified the direct and indirect effects of multiple environmental factors, such as temperature and trace elements, on the zooplankton community in mountain rivers and has revealed the complex interaction relationships among them. It provided a new perspective for understanding the ecological processes in mountain river ecosystems. Future research should conduct long-term monitoring, incorporate more variables, and deeply study the interactive effects of multiple environmental factors to further improve our understanding of the zooplankton community in mountain rivers and provide a more solid scientific basis for ecological protection and resource management.

## Data Availability

The data that support the findings of this study are available from the corresponding author or the first author upon reasonable request.

## Supplementary Materials

The following supporting information can be downloaded at https://www.mdpi.com/article/10.3390/biology14020183/s1, Figure S1: Alpha diversity of zooplankton taxa; Figure S2: Hierarchical partitioning (HP) analysis. The values as percentages (%) indicate the independent contribution of each of the 29 environmental variables; Table S1: The transparency of the Taizicheng River over four months. The unit is centimeters (cm); Table S2: Correlations of zooplankton biomass and diversities with environmental factors from the Mantel test based on the Spearman rank correlation.
