# Supplementary material for: Trace Elements and Temperature Combined to Regulate Zooplankton Community Structures in Mountain Streams
Source: Biology (Basel). 2025 Feb 11;14(2):183. doi: 10.3390/biology14020183 (PMC11851842; doi:10.3390/biology14020183)
Supplement: Supplementary file 1 [file biology-14-00183-s001.zip › biology-3466668-supplementary.pdf]

Trace Elements and Temperature Combined to Regulate Zooplankton Community Structures in Mountain Streams

Li Ji [1], Huayong Zhang [1,2,*], Zhongyu Wang [1], Yonglan Tian [1], Wang Tian [1] and Zhao Liu [2]

1 Research Center for Engineering Ecology and Nonlinear Science, North China Electric Power University, Beijing 102206, China; geelyz@163.com (L.J.); zhy_wang@ncepu.edu.cn (Z.W.); yonglantian@ncepu.edu.cn (Y.T.); tianwang@ncepu.edu.cn (W.T.)

2 Theoretical Ecology and Engineering Ecology Research Group, School of Life Sciences, Shandong University, Qingdao 250100, China; liuzhao9555@sdu.edu.cn

* Correspondence: zhanghuayong@sdu.edu.cn

Supplementary Figures

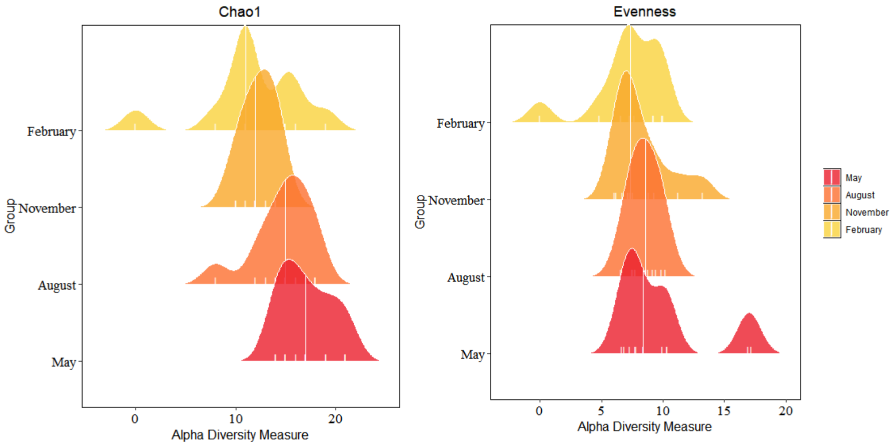

Figure S1 Alpha diversity of zooplankton taxa.

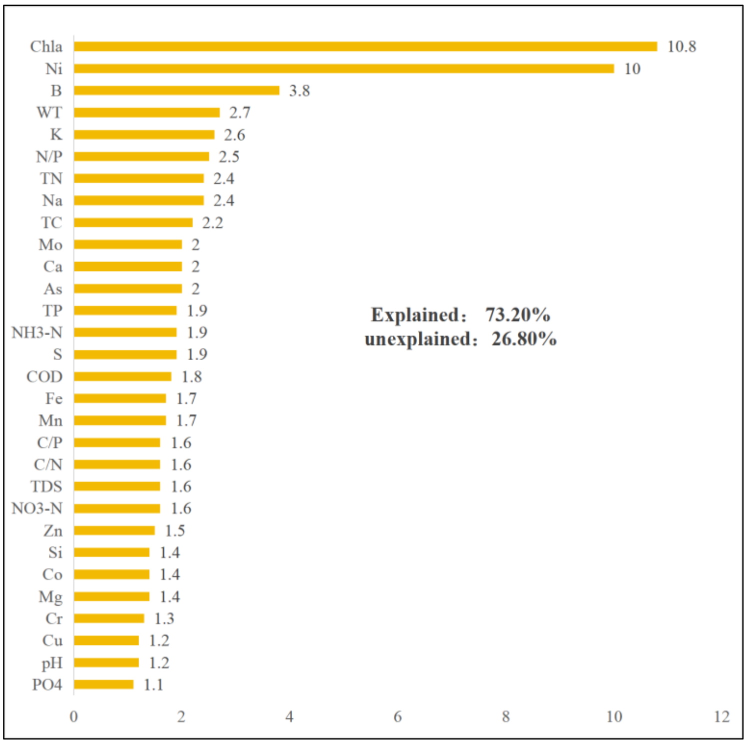

Figure S2 Hierarchical partitioning (HP) analysis. The values as percentage (%) indicate the independent contribution of each of 29 environmental variables.

Supplementary Tables

Table S1 The transparency of Taizicheng river under four months. The unit is centimeters (cm).

	May	August	November	February
T02	12	3	22	22
T04	6	5	12	10
T05	8	2	10	8
T06	13	2	8	2
T07	12	4	7	10
T08	13	2	8	8
T09	70	36	10	11
T10	16	15	13	4
Z01	10	9	14	11
Z02	70	10	13	14
Z03	30	10	1	15

Table S2 Correlations of zooplankton biomass and diversities with environmental factors from Mantel test based on spearman rank correlation.

Zooplankton	Environmental factors	r	p	Mantel's r	Mantel's p
Biomass	WT	0.153338748	0.008	< 0.2	< 0.01
	pH	0.014293537	0.413	< 0.2	>= 0.05
	COD	-0.143375607	0.963	< 0.2	>= 0.05
	Chla	0.137267434	0.064	< 0.2	>= 0.05
	TDS	-0.073640182	0.818	< 0.2	>= 0.05
	TC	-0.030322259	0.628	< 0.2	>= 0.05
	TN	-0.017747758	0.571	< 0.2	>= 0.05
	TP	-0.07919479	0.817	< 0.2	>= 0.05
	C/N	0.016754071	0.412	< 0.2	>= 0.05
	N/P	0.014450272	0.392	< 0.2	>= 0.05
	C/P	-0.009325946	0.427	< 0.2	>= 0.05
	PO_4	-0.066706056	0.749	< 0.2	>= 0.05
	NH_3-N	0.003859677	0.429	< 0.2	>= 0.05
	NO_3-N	0.043293152	0.251	< 0.2	>= 0.05
	S	-0.033915944	0.641	< 0.2	>= 0.05
	Na	0.22281853	0.002	0.2 - 0.4	< 0.01
	Ca	0.028070989	0.327	< 0.2	>= 0.05
	Mg	-0.013215061	0.491	< 0.2	>= 0.05
	Cr	-0.070940106	0.679	< 0.2	>= 0.05
	Fe	-0.01866417	0.529	< 0.2	>= 0.05
	Mn	0.040273018	0.332	< 0.2	>= 0.05
	Co	0.078340864	0.133	< 0.2	>= 0.05
	Cu	-0.020325298	0.546	< 0.2	>= 0.05
	Zn	-0.02986032	0.518	< 0.2	>= 0.05
	K	0.137018689	0.051	< 0.2	>= 0.05
	Si	0.062977392	0.256	< 0.2	>= 0.05
	Ni	0.297260775	0.001	0.2 - 0.4	< 0.01
	As	-0.04459639	0.694	< 0.2	>= 0.05
	Mo	0.14749299	0.02	< 0.2	0.01 - 0.05
	B	-0.02428612	0.622	< 0.2	>= 0.05
observed	WT	0.234447953	0.001	0.2 - 0.4	< 0.01
	pH	0.110634647	0.12	< 0.2	>= 0.05
	COD	-0.044630039	0.639	< 0.2	>= 0.05
	Chla	0.015375055	0.385	< 0.2	>= 0.05
	TDS	0.089806368	0.131	< 0.2	>= 0.05
	TC	0.050572029	0.236	< 0.2	>= 0.05
	TN	0.00086304	0.457	< 0.2	>= 0.05
	TP	0.117438727	0.105	< 0.2	>= 0.05
	C/N	0.066825972	0.161	< 0.2	>= 0.05
	N/P	0.013665653	0.356	< 0.2	>= 0.05

	C/P	0.081944562	0.152	< 0.2	>= 0.05
	PO_4	0.113771711	0.116	< 0.2	>= 0.05
	NH_3-N	-0.004063601	0.403	< 0.2	>= 0.05
	NO_3-N	-0.066777917	0.834	< 0.2	>= 0.05
	S	0.075302791	0.152	< 0.2	>= 0.05
	Na	0.114804824	0.026	< 0.2	0.01 - 0.05
	Ca	-0.020026406	0.553	< 0.2	>= 0.05
	Mg	-0.052990405	0.678	< 0.2	>= 0.05
	Cr	-0.082031778	0.813	< 0.2	>= 0.05
	Fe	0.108320272	0.114	< 0.2	>= 0.05
	Mn	0.008281962	0.411	< 0.2	>= 0.05
	Co	-0.024468811	0.603	< 0.2	>= 0.05
	Cu	0.078667329	0.179	< 0.2	>= 0.05
	Zn	-0.018275482	0.455	< 0.2	>= 0.05
	K	0.004066133	0.423	< 0.2	>= 0.05
	Si	0.132196889	0.078	< 0.2	>= 0.05
	Ni	0.185903835	0.001	< 0.2	< 0.01
	As	0.082956864	0.168	< 0.2	>= 0.05
	Mo	0.003714274	0.408	< 0.2	>= 0.05
	B	0.14760938	0.022	< 0.2	0.01 - 0.05
Shannon	WT	0.052474506	0.168	< 0.2	>= 0.05
	pH	-0.05938554	0.692	< 0.2	>= 0.05
	COD	0.037918267	0.284	< 0.2	>= 0.05
	Chla	0.058261675	0.227	< 0.2	>= 0.05
	TDS	-0.070100178	0.794	< 0.2	>= 0.05
	TC	0.053498061	0.26	< 0.2	>= 0.05
	TN	0.114847599	0.074	< 0.2	>= 0.05
	TP	0.115413203	0.109	< 0.2	>= 0.05
	C/N	0.007442722	0.385	< 0.2	>= 0.05
	N/P	-0.045109384	0.554	< 0.2	>= 0.05
	C/P	-0.059911105	0.689	< 0.2	>= 0.05
	PO_4	0.125491812	0.117	< 0.2	>= 0.05
	NH_3-N	0.339670395	0.021	0.2 - 0.4	0.01 - 0.05
	NO_3-N	-0.036282445	0.616	< 0.2	>= 0.05
	S	0.007463073	0.406	< 0.2	>= 0.05
	Na	0.045157281	0.183	< 0.2	>= 0.05
	Ca	0.044479671	0.231	< 0.2	>= 0.05
	Mg	0.073618208	0.18	< 0.2	>= 0.05
	Cr	-0.066032281	0.694	< 0.2	>= 0.05
	Fe	0.122950795	0.107	< 0.2	>= 0.05
	Mn	0.129415054	0.121	< 0.2	>= 0.05
	Co	-0.037836592	0.644	< 0.2	>= 0.05
	Cu	-0.013091019	0.448	< 0.2	>= 0.05

	Zn	-0.024657036	0.39	< 0.2	>= 0.05
	K	0.070044868	0.192	< 0.2	>= 0.05
	Si	0.034541035	0.254	< 0.2	>= 0.05
	Ni	0.051510018	0.092	< 0.2	>= 0.05
	As	0.045604068	0.265	< 0.2	>= 0.05
	Mo	-0.009271521	0.479	< 0.2	>= 0.05
	B	0.069835696	0.146	< 0.2	>= 0.05
